# Morbility, clinical data and proteomic analysis of IUGR and AGA newborns at different gestational ages

**DOI:** 10.1016/j.dib.2016.09.024

**Published:** 2016-09-21

**Authors:** M.D. Ruiz-González, M.D. Cañete, J.L. Gómez-Chaparro, A. Rodríguez-Torronteras, N. Abril, R. Cañete, J.L. López-Barea

**Affiliations:** a Neonatology Unit, Pediatrics Service, Reina Sofía University Hospital, Andalusian Health Service, Menéndez Pidal Avenue, 14004 Córdoba, Spain; b Maimonides Institute of Biomedical Research of Córdoba (IMIBIC), Menendez Pidal Avenue, 14004 Córdoba, Spain; c Experimental Unit Córdoba District, Andalusian Health Service, Isla Lanzarote s/n, 14011 Córdoba, Spain; d Epidemiology Unit Córdoba District, Andalusian Health Service, Isla Lanzarote s/n, 14011 Córdoba, Spain; e Department of Biochemistry and Molecular Biology, Severo Ochoa Building, Rabanales Campus, University of Córdoba, A4 highway, Km 396a, 14071 Córdoba, Spain; f Pediatric Endocrinology Section, Pediatrics Service, Reina Sofía University Hospital, Andalusian Health Service, Menéndez Pidal Avenue, 14004 Córdoba, Spain

**Keywords:** Human newborn, Intrauterine growth restriction, Venous blood, Serum proteins, Proteomics

## Abstract

The data are related to the proteomic analysis of 43 newborns with intrauterine growth retardation (IUGR) and 45 newborns with appropriate weight for gestational age (AGA) carried out by separation via 2DE and analyzed by MS–TOF/TOF. All newborns were separated into three gestational age groups, "Very Preterm" 29–32 weeks, "Moderate Preterm" 33–36 weeks, and, "Term" ≥37weeks. From each newborn, blood was drawn three times from birth to 1 month life. High-abundant serum proteins were depleted, and the minority ones were separated by 2DE and analyzed for significant expression differences. The data reflect analytic and clinic variables analyzed globally and categorized by gestational age in relation to IUGR and the optimization of conditions for 2-DE separation. The data from this study are related to the research article entitled "Alterations of Protein Expression in Serum of Infants with Intrauterine Growth Restriction and Different Gestational Ages" (M.D. Ruis-González, M.D. Cañete, J.L. Gómez-Chaparro, N. Abril, R. Cañete, J. López-Barea, 2015) [1]. The present dataset of serum IUGR newborn proteome can be used as a reference for any study involving intrauterine growth restriction during the first month of life.

**Specifications Table**

**Table:** 

Subject area	*Biology*
More specific subject area	*Neonatology, IUGR (intrauterine growth restriction)*
Type of data	*Tables and figures*
How data was acquired	*By clinical history and 2-DE gel PAGE*
Data format	*Analysed*
Experimental factors	*Samples were subjected to protein depletion using the Proteominer*^*TM*^*kit (Bio-Rad*^*®)*^*_prior to separation by 2-DE electrophoresis_*
Experimental features	*Standard procedures of laboratory and 2-DE gels electrophoresis*
Data source location	*Córdoba (Spain)*
Data accessibility	*The data are supplied with this article*

**Value of the data**

• The clinical characteristics of IUGR neonates, and the statistical analysis of quantitative and qualitative variables, carried out both globally and in function of gestational age can be useful for other scientists working in the IUGR field.
• The set-up and optimization of the conditions of 2-DE analysis in sera from newborns could be useful for the reproducibility of similar future trials.
• This dataset could be used as a benchmark for studies on the variations of serum proteomic IUGR from birth through the first month of life.

## 1 Data

Descriptive analysis of analytical and clinical variables was carried out globally (Table 1, Table 2, Table 3) and categorized also according to gestational age (GA) groups in “Very Preterm”, “Moderate Preterm” and “Term” IUGR infants at different times after birth (0–48 h, 7–10d, 28–30d) (Table 4, Table 5, Table 6). Table 7 illustrates the pathogenic microorganisms isolated in positive hemocultures.

**Fig. 1:**
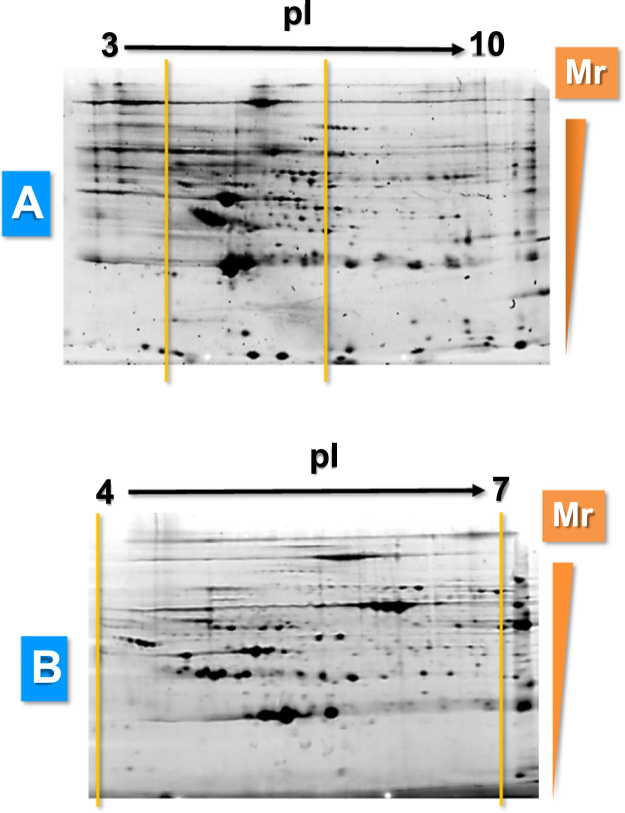
Representative gels obtained for optimization of 2DE separation of high-abundance depleted human serum proteins via IEF with IPG Strips (11 cm) of pH 3–10 (A) or pH 4–7 (B).

For the development of 2-DE electrophoresis, we started carrying our 2-DE gels with a wide pH range (3–10), as shown in Fig. 1A. In these initial gels we observed that most of the proteins were in the pH range 4–7. Thus, we reasoned that the resolution would improve using IPG strips of narrower pH gradient. Thus, 2-DE electrophoresis was routinely carried out using IPG strips of 11 cm and pH range 4–7, as shown in Fig. 1B. Isoelectric focusing was performed using an Protean IEF Cell (BioRad) a 20 °C according the next program (Table 8).

## 2 Experimental design, materials and methods

### 2.1 Subject selection

We included IUGR neonates, defined by having a birth weight <10th centile for gestational age (GA), according to Carrascosa curves [2], together with echographic evidence of altered placental function, identified as an abnormal Doppler of the umbilical artery, or reduced growth rate [3]. Also included were 45 infants with a birth weight appropriate for their gestational age (AGA), paired for sex and classified in 3 groups by their GA [1].

### 2.2 Statistical analysis

The descriptive analysis of the variables studied was carried out with the routine methodology. Comparison of variables between the RCIU and AGA groups was carried out using the Student *t*-test for quantitative variables and the *χ*^2^-test for qualitative variables. The Fisher exact test was applied when conditions of applicability of the *χ*^2^-test were not fulfilled. A value of *p*<0.05 was established as statistically significant. The R statistical software, version 3.22, was used throughout the whole study [4].

## Figures and Tables

**Table 1:** Global analysis of qualitative variables analyzed in IUGR neonates.

**Variable**	**Infant type**	***N***	***n* (%)**	***P*****value**
**Respiratory dystres**	IUGR	43	13 (30.2)	0.171
	AGA	45	8 (17.8)	
				
**NEC**	IUGR	43	2 (4.7)	0.143
	AGA	45	0	
				
**Hypoxia-ischemia**	IUGR	43	0	NA
	AGA	45	0	
				
**Hypoglycemia 0–48 h**	IUGR	43	13 (30.2)	0.171
	AGA	45	8 (17.8)	
				
**Hypoglycemia 7–10d**	IUGR	43	1 (2.3)	0.974
	AGA	45	1 (2.2)	
				
**Primiparae**	IUGR	43	30 (69.8)	0.166
	AGA	45	30 (66.7)	
				
**Prenatal corticosteroids**	IUGR	43	22 (51.2)	0.839
	AGA	45	24 (53.3)	
				
**Preeclampsia**	IUGR	43	13 (30.2)	⁎**0.001**
	AGA	45	2 (4.4)	
				
**Pregnancy-induced hypertension**	IUGR	43	6 (13.9)	⁎**0.001**
	AGA	45	1 (2.2)	
				
**Oligohydramnios**	IUGR	43	3 (7.0)	0.079
	AGA	45	0	
				
**Thrombocytopenia 0–48 h**	IUGR	43	8 (18.6)	⁎**0.002**
	AGA	45	0	
				
**Thrombocytopenia 7–10d**	IUGR	43	4 (9.3)	⁎**0.036**
	AGA	45	0	
				
**Thrombocytopenia 28–30d**	IUGR	43	2 (4.7)	0.143
	AGA	45	0	
				
**Hemocultive + 0–48 h**	IUGR	43	1 (2.3)	0.328
	AGA	45	3 (6.7)	
				
**Hemocultive + 7–10d**	IUGR	43	11 (25.6)	⁎**0.005**
	AGA	45	2 (4.4)	
				
**Hemocultive + 28–30d**	IUGR	43	2 (4.7)	0.143
	AGA	45	0	
				
**Polycythaemia 0–48 h**	IUGR	43	2 (4.7)	0.143
	AGA	45	0	
				
**Smoking**	IUGR	43	1 (2.3)	0.304
	AGA	45	0	
				
**Alcohol/other drugs**	IUGR	43	0	NA
	AGA	45	0	

**IUGR**. intrauterine growth restriction; **AGA**. appropriate for gestational age; **NEC**. necrotizing enterocolitis; **NA**. not applicable

⁎ significant *(p* < 0.05)

**Table 2:** Global analysis of qualitative variables (16) analyzed in IUGR neonates of the three gestational age groups.

**Variable**	**Infant type**	***N***	**Group 1 (GA 29 a 32w)**	***P***	***N***	**Group 2 (GA 33 a 36w)**	***P***	***N***	**Group 3 (GA ≥ 37 w)**	***P***
**Respiratory dystres**	IUGR	13	6 (46.15)	0.488	15	4 (26.66)	0.142	15	3 (20.00)	0.624
	AGA	15	5 (33.33)		15	1 (06.66)		15	2 (13.00)	
										
**NEC**	IUGR	13	–	NA	15	2 (13.00)	0.143	15	–	NA
	AGA	15	–		15	0		15	–	
										
**Hypoxia-Ischemia**	IUGR	13	–	NA	15	–	NA	15	–	NA
	AGA	15	–		15	–		15	–	
										
**Hypoglycemia 0–48 h**	IUGR	13	2 (15.38)	0.150	15	3 (20.00)	0.624	15	8 (53.33)	⁎**0.001**
	AGA	15	6		15	2 (13.00)		15	0	
										
**Hypoglycemia 7–10d**	IUGR	13	–	NA	15	1 (06.66)	0.974	15	–	NA
	AGA	15	–		15	1 (06.66)		15	–	
										
**Primiparae**	IUGR	13	9 (69.23)	0.229	15	9 (60.00)	0.512	15	12 (80.00)	0.512
	AGA	15	7 (46.66)		15	10 (66.7)		15	13 (86.6)	
										
**Prenatal corticosteroids**	IUGR	13	12 (92.3)	0.630	15	9 (60.00)	0.439	15	1 (06.66)	0.309
	AGA	15	13 (86.7)		15	11 (73.3)		15	0	
										
**Preeclampsia**	IUGR	13	6 (46.15)	0.055	15	7 (46.66)	⁎**0.003**	15	–	NA
	AGA	15	2 (13.33)		15	0		15	–	
										
**PIH**	IUGR	13	3 (23.07)	0.216	15	1 (06.66)	0.309	15	2 (13.33)	0.143
	AGA	15	1 (06.66)		15	0		15	0	
										
**Oligohydramnios**	IUGR	13	2 (07.69)	0.115	15	–	NA	15	1 (06.66)	0.309
	AGA	15	0		15	–		15	0	
										
**Thrombocytopenia 0–48 h**	IUGR	13	2 (07.69)	0.115	15	5 (33.33)	⁎**0.014**	15	1 (06.66)	0.309
	AGA	15	0		15	0		15	0	
										
**Thrombocytopenia 7–10d**	IUGR	13	1 (07.69)	0.274	15	2 (13.33)	0.143	15	1 (07.69)	0.309
	AGA	15	0		15	0		15	0	
										
**Thrombocytopenia 28–10d**	IUGR	13	–	NA	15	1 (07.69)	0.309	15	–	NA
	AGA	15	–		15	0		15	–	
										
**Hemocultive + 7–10d**	IUGR	13	7 (53.84)	⁎**0.022**	15	4 (26.66)	⁎**0.032**	15	–	NA
	AGA	15	2 (13.33)		15	0		15	–	
										
**Polycythaemia 0–48 h**	IUGR	13	–	NA	15	1	0309	15		0.309
	AGA	15	–		15	0		15	0	
										
**Smoking**	IUGR	13	1 (07.69)	0.274	15	–	NA	15	–	NA
	AGA	15	0		15	–		15	–	

**IUGR**. intrauterine growth restriction; **AGA**. appropriate for gestational age; **NEC**. necrotizing enterocolitis; **PIH**. pregnancy-induced hypertension; **NA**. not applicable.

⁎ significant *(p* < 0.05).

**Table 3:** Global descriptive analysis of quantitative variables (20) analyzed in IUGR neonates and AGA infants along the three sampling times.

**Variable**	**Infant type**	**Sampling time 0–48 h**			**Sampling time 7–10 days**			**Sampling time 28–30 days**		
		***N***	**Value**	***P***	***N***	**Value**	***P***	***N***	**Value**	***P***
**RCP (mg/L)**	IUGR	40	01.39±2.78	**^⁎^0.017**	33	07.00±17.3	0.313	18	06.92±14.7	0.052
	AGA	39	12.25±2.79		37	03.57±10.3		25	01.21±2.28	
										
**PCT (ng/mL)**	IUGR	8	00.21±0.21	0.263	14	01.70±3.10	0.316	3	04.72±7.00	0.624
	AGA	19	09.25±2.20		6	00.36±0.47		1	0.08------	
										
**Hb (g/dL)**	IUGR	43	18.15±2.02	**^⁎^<0.001**	40	15.72±2.45	**^⁎^0.009**	37	10.88±1.84	0.528
	AGA	45	15.57±2.13		41	14.19±2.62		42	10.56±2.01	
										
**Hto (%)**	IUGR	43	54.39±6.33	**^⁎^<0.001**	40	47.66±7.36	**^⁎^0.008**	38	32.02±5.19	0.814
	AGA	45	47.90±5.95		41	42.99±8.13		42	31.71±6.15	
										
**Leukocytes (10**^**3**^**/µL)**	IUGR	43	13,416±7659	0.151	40	12,184±4253	0.543	39	10,804±3305	0.569
	AGA	45	21,036±3327		41	12,849±5465		42	11,224±3298	
										
**Neutrophils (%)**	IUGR	43	49.67±15.5	0.558	40	35.33±12.0	**^⁎^0.018**	38	23.88±8.39	0.502
	AGA	45	51.64±15.6		41	41.58±11.3		42	25.14±8.39	
										
**Lymphocyte (%)**	IUGR	43	39.41±15.1	0.204	40	43.63±12.3	0.202	39	55.57±10.0	0.222
	AGA	45	35.34±14.3		41	40.23±11.5		41	58.24±19.3	
										
**Monocytes (%)**	IUGR	43	07.56±3.81	0.129	40	14.75±5.38	**^⁎^0.037**	39	09.31±2.58	0.595
	AGA	45	08.94±4.44		41	12.32±4.91		42	08.96±3.27	
										
**Eosinophils (%)**	IUGR	43	02.46±1.88	0.785	40	04.34±2.93	0.719	39	06.92±5.05	0.111
	AGA	45	02.36±1.59		41	04.14±1.92		42	05.40±3.34	
										
**Basophils (%)**	IUGR	43	00.84±0.55	0.196	40	01.37±0.80	0.110	36	00.89±0.46	0.442
	AGA	45	01.17±1.46		41	01.11±0.63		42	00.81±0.39	
										
**Platelets (10**^**3**^**/µL)**	IUGR	43	181,785± 61,103	**^⁎^<0.001**	40	273,275± 124,266	**^⁎^0.001**	39	380,435± 124,497	0.761
	AGA	45	266,622± 83,951		41	363,170± 111,290		42	390,785± 174,925	
										
**AST (U/L)**	IUGR	30	70.00±86.0	0.814	34	35.00±18.0	0.428	37	35.0±40.0	0.402
	AGA	26	66.00±44.0		37	31.00±14.0		37	30.0±12.0	
										
**ALT (U/L)**	IUGR	30	25.00±48.0	0.959	32	14.00±17.0	0.410	25	18.0±5.00	0.052
	AGA	26	26.00±49.0		37	19.00±26.0		24	20.0±9.00	
										
**Glucose (mg/dL)**	IUGR	42	51.00±32.0	0.525	40	79.00±26.0	0.399	42	81.00±18.0	0.534
	AGA	44	52.0±36.0		44	84.00±24.0		43	78.00±12.0	
										
**Protein (g/L)**	IUGR	43	05.00±0.75	0.524	39	05.20±0.55	0.199	42	04.68±0.85	0.191
	AGA	45	05.10±1.06		44	05.39±0.76		44	04.90±0.65	
										
**K (mEq/L)**	IUGR	42	04.59±0.81	0.532	39	04.80±0.78	0.674	40	05.28±0.69	0.813
	AGA	43	04.70±0.78		44	04.72±0.78		44	05.25±0.65	
										
**Na (mEq/L)**	IUGR	45	135.0±3.29	0.171	40	135.6±3.76	0.183	41	136.2±2.33	0.899
	AGA	45	136.2±3.29		44	136.6±3.31		44	136.1±3.33	
										
**Ca (mg/dL)**	IUGR	40	10.26±0.88	0.239	39	11.14±0.57	0.259	31	11.00±0.80	0.051
	AGA	41	10.02±0.93		43	11.00±0.54		39	11.32±0.50	
										
**Urea (mg/dL)**	IUGR	43	24.65±13.93	0.421	40	18.15±14.1	0.634	42	13.80±9.13	0.776
	AGA	41	26.90±11.40		44	19.61±14.8		44	13.31±6.60	
										
**Cre (mg/dL)**	IUGR	42	00.69±0.19	0.449	40	00.48±0.14	0.473	41	00.38±0.06	0.511
	AGA	40	00.73±0.26		45	00.51±0.12		44	00.37±0.07	

**IUGR**. intrauterine growth restriction; **AGA**. appropriate for gestational age; **RCP**. reactive C protein; **PCT**. procalcitonin; **Hb**. hemoglobin; **AST**. aspartate aminotransferase; **K**. potassium; **Na**. sodium; **Cre**. creatinine.

**Table 4:** Descriptive analysis categorized according to the gestational ages of quantitative variables of "Very Preterm" IUGR neonates at different times after birth.

**Group 1 (GA 29 to 32w)**										
**Variable**	**Infant type**	***N***	**0-48h**	***P***	***N***	**7–10d**	***P***	***N***	**28–30d**	***P***
**RCP (mg/L)**	IUGR	13	00.93±1.40	0.279	13	11.09±22.1	0.493	11	06.95±11.5	0.468
	AGA	12	04.19±10.5		13	05.66±17.3		7	02.18±3.72	
										
**Hb (g/dl)**	IUGR	13	17.36±1.66	⁎**0.012**	13	14.17±2.55	0.265	11	10.60±1.93	0.588
	AGA	15	15.67±1.64		13	13.04±2.49		14	10.22±1.93	
										
**Hto (%)**	IUGR	13	51.66±5.49	⁎**0.028**	13	42.80±7.82	0.157	11	31.86±5.24	0.647
	AGA	15	47.34±4.25		13	38.63±6.68		14	30.87±5.28	
										
**Platelets (10**^**3**^**/µL)**	IUGR	13	196,307± 52,948	⁎**0.008**	13	242,230± 104,620	0.029	12	369,833± 143,023	0.523
	AGA	15	261,000± 64,583		13	340,769± 112,243		14	409,000± 161,771	
										
**PMN (%)**	IUGR	13	34.40±10.4	0.498	13	38.01±16.4	0.231	12	24.01±10.3	0.816
	AGA	15	38.38±12.2		13	45.50±14.6		14	24.82±7.16	
										
**Monocytes (%)**	IUGR	13	08.71±5.61	0.434	13	18.16±7.36	⁎**0.008**	12	09.15±2.33	0.392
	AGA	15	10.62±6.73		13	10.89±5.40		11	09.94±2.23	

**GA**: gestational age; **IUGR**: intrauterine growth restriction; **RCP**: reactive C protein; **Hb**: hemoglobin; **Hto**: Hematocrit; **PMN**: polymorphonuclears.

⁎ significant (*p* < 0.05)

**Table 5:** Descriptive analysis categorized according to the gestational ages of quantitative variables of "Moderate Preterm" IUGR neonates at different times after birth.

**Group 2 (GA 33 to 36w)**										
**Variable**	**Infant type**	***N***	**0-48h**	***P***	***N***	**7–10d**	***P***	***N***	**28–30d**	***P***
**RCP (mg/L)**	IUGR	15	01.94±4.11	0.157	9	08.36±19.65	0.203	5	09.54±11.23	0.115
	AGA	13	00.26±0.28		11	00.58±0.79		6	01.41±2.29	
										
**Hb (g/dl)**	IUGR	15	18.26±1.98	⁎**0.001**	12	16.09±1.84	⁎**0.003**	12	10.59±1.99	0.112
	AGA	15	15.13±2.19		14	13.47±2.19		14	09.41±1.87	
										
**Hto (%)**	IUGR	15	54.24±6.07	⁎**0.002**	12	48.63±5.27	⁎**0.001**	15	30.92±5.75	0.275
	AGA	15	45.95±7.00		14	40.15±5.79		14	28.52±5.81	
										
**Platelets (10**^**3**^**/µL)**	IUGR	15	170,866± 73,647	⁎**0.001**	12	300,166± 133,091	⁎**0.030**	15	382,866± 196,369	0.312
	AGA	15	303,333± 117,968		14	417,571± 125,444		14	450,142± 232,764	
										
**Neutrophils (%)**	IUGR	15	50.06±12.66	0.681	12	34.65±12.07	0.230	14	25.03±9.08	0.316
	AGA	15	51.18±12.17		14	39.44±7.51		14	28.66±9.70	
										
**Monocytes (%)**	IUGR	15	06.72±2.55	0.683	12	12.31±2.50	0.458	15	08.20±2.06	0.620
	AGA	15	07.10±2.28		14	13.12±3.29		15	08.81±3.70	

**GA**: gestational age; **IUGR**: intrauterine growth restriction; **RCP**: reactive C protein; **Hb**: hemoglobin; **Hto**: Hematocrit

⁎ significant (*p* < 0.05)

**Table 6:** Descriptive analysis categorized according to the gestational ages of quantitative variables of "Term" IUGR neonates at different times after birth.

**Group 3 (GA ≥ 37s w)**										
**Variable**	**Infant type**	***N***	**0–48 h**	***P***	***N***	**7–10d**	***P***	***N***	**28–30d**	***P***
**RCP (mg/L)**	IUGR	12	01.19±1.69	⁎**0.021**	11	01.06±1.93	⁎**0.009**	2	00.20±1.93	0.493
	AGA	14	30.20±4.48		13	04.02±2.92		12	00.54±2.92	
										
**Hb (g/dl)**	IUGR	14	18.76±2.26	⁎**0.002**	15	16.78±2.24	0.439	11	11.42±1.55	0.351
	AGA	15	15.92 ±2.25		13	16.11±2.21		14	12.06±1.73	
										
**Hto (%)**	IUGR	14	57.12±6.57	⁎**0007**	15	51.09±6.43	0.634	12	33.53±4.39	0.264
	AGA	15	50.43±5.73		14	49.88±7.05		14	35.75±5.33	
										
**Platelets (10**^**3**^**/µL)**	IUGR	14	180,000± 54,721	⁎**0.004**	15	278,666± 134,803	0.226	12	388,000± 145,174	0.104
	AGA	15	235,533± 39,362		14	329,571± 76,415		14	313,214± 74,313	
										
**Neutrophils (%)**	IUGR	14	62.52±10.4	0.434	14	33.54±6.80	0.060	12	22.40±5.34	0.861
	AGA	15	65.38±8.98		15	40.10±10.9		14	21.95±7.23	
										
**Monocytes (%)**	IUGR	14	06.72±2.55	0.683	15	12.31±2.50	0.458	15	08.20±2.06	0.620
	AGA	15	07.10±2.28		14	13.12±3.29		14	08.81±3.29	

**GA**: gestational age; **IUGR**: intrauterine growth restriction; **RPC**: reactive C protein; **Hb**: hemoglobin; **Hto**: Hematocrit.

⁎ significant (*p* < 0.05)

**Table 7:** Pathogenic microorganisms isolated in hemocultures.

**Germ**	**Hemocultures number**
*Staphylococcus epidermidis*	10
*Staphylococcus aureus*	1
*Staphylococcus scheleiferi*	1
*Staphylococcus haemolyticus*	1
*Staphylococcus auricularis*	2
*Staphylococcus hominis-hominis*	1
*Serratia marcenscens*	1
*Klebsiella pneumoniae*	1
*Candida parapsilopsis*	1

**Table 8:** IEF program conducted Protean IEF Cell system (Bio-Rad).

**Stage**	**Voltage (V)**	**Duration (hours:minutes)**	**Ramp**
Passive rehydration	–	6:00	–
Active rehydration	50	6:00	Rapid
Phase 1	500	1:00	Lineal
Phase 2	1.000	1:00	Lineal
Phase 3	3.000	1:00	Lineal
Phase 4	6.000	2:00	Lineal
Phase 5	6.000	1:00	Rapid
